# Oesophageal Epithelial Cell‐Intrinsic MHCII Regulates Food Antigen‐Dependent Eosinophilic Esophagitis in an IFNγ‐Dependent Manner

**DOI:** 10.1111/cea.70205

**Published:** 2025-12-22

**Authors:** Eric M. Rodríguez‐López, Rachel L. Clement, Megha Lal, Yusen Zhou, Stephen D. Carro, Charles‐Antoine Assenmacher, Ravi Gautam, Jarad Beers, Amanda B. Muir, Jonathan M. Spergel, Melanie A. Ruffner, Laurence C. Eisenlohr, David A. Hill

**Affiliations:** ^1^ Institute for Immunology and Immune Health, Perelman School of Medicine University of Pennsylvania Philadelphia Pennsylvania USA; ^2^ Immunology Graduate Group, Perelman School of Medicine University of Pennsylvania Philadelphia Pennsylvania USA; ^3^ Division of Allergy and Immunology Children's Hospital of Philadelphia Philadelphia Pennsylvania USA; ^4^ Department of Biomedical and Health Informatics Children's Hospital of Philadelphia Philadelphia Pennsylvania USA; ^5^ Cell and Molecular Biology Graduate Group, Perelman School of Medicine University of Pennsylvania Philadelphia Pennsylvania USA; ^6^ Department of Pathology and Laboratory Medicine Children's Hospital of Philadelphia Philadelphia Pennsylvania USA; ^7^ Comparative Pathology Core, School of Veterinary Medicine University of Pennsylvania Philadelphia Pennsylvania USA; ^8^ Division of Gastroenterology, Hepatology and Nutrition Children's Hospital of Philadelphia Philadelphia Pennsylvania USA; ^9^ Department of Pediatrics, Perelman School of Medicine University of Pennsylvania Philadelphia Pennsylvania USA

**Keywords:** eosinophilic esophagitis, food allergy, immunoregulation, interferon gamma, major histocompatibility complex class II, oesophageal epithelial cells

## Abstract

**Background:**

Eosinophilic oesophagitis (EoE) is a chronic food allergy that causes oesophageal inflammation and dysfunction. Recent work demonstrates IFNγ‐dependent gene signatures in inflamed EoE biopsies. IFNγ has been implicated in the promotion of MHCII expression on oesophageal epithelial cells (EECs). However, the regulation of EEC‐MHCII expression in vivo, and its contribution to EoE, is unknown.

**Objective:**

The objective of this study was to determine the regulation and role of EEC‐intrinsic MHCII expression in EoE.

**Methods:**

We examined the expression of HLA II‐pathway transcripts in human EECs using single cell RNA‐seq datasets and primary human tissues and mouse systems to interrogate the contribution of IFNγ to EEC‐MHCII expression. Finally, we used a mouse disease model to test the contribution of epithelial MHCII to food antigen‐dependent EoE.

**Results:**

HLA II transcripts were upregulated in EECs of active EoE patients, compared with controls. Similarly, EEC‐MHCII expression was higher in mice with EoE‐like inflammation. EEC‐MHCII expression was governed by IFNγ‐responsive transcriptional regulation. EEC‐specific MHCII deficiency resulted in exacerbated eosinophilic inflammation in a model of food antigen‐dependent EoE.

**Conclusion:**

We find a novel immunoregulatory role for IFNγ‐dependent EEC‐MHCII in the context of oesophageal food allergy.

**Clinical Relevance:**

Our results expand our understanding of oesophageal immune physiology and identify EEC‐MHCII as mediating an anti‐inflammatory axis that could be leveraged therapeutically.

## Summary

- Oesophageal epithelial cells upregulate MHCII expression during active EoE.
- MHCII expression by EECs is governed by IFNγ‐inducible transcriptional elements.
- EEC‐intrinsic MHCII attenuates eosinophilic oesophageal inflammation.

## Graphical abstract

We found that oesophageal epithelial cells upregulate MHCII expression during active EoE in humans and a mouse model. MHCII expression by EECs was governed by IFNγ‐inducible transcriptional elements. EEC‐intrinsic MHCII promoted regulatory T cell accumulation and attenuated eosinophilic oesophageal inflammation.

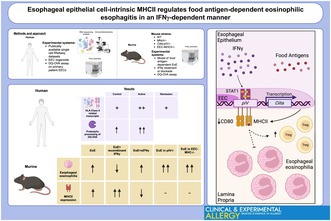

## 1 Introduction

Eosinophilic oesophagitis (EoE) is a chronic allergic disease that is characterised by eosinophilic inflammation in the oesophagus, disruption of oesophageal barrier integrity, and ultimately fibrosis and stricture formation. The result is oesophageal dysfunction that can present as abdominal pain, dysphagia, and/or reflux‐like symptoms. If left untreated, severe inflammation and oesophageal remodelling can predispose to food impactions [1].

Research performed by a variety of groups has described EoE as predominantly a type 2 (T2) inflammatory process, as multiple immune cells and cytokines associated with these pathways have been implicated in its immunopathology [2, 3]. Indeed, the success of dupilumab, an FDA‐approved anti‐IL4Ra monoclonal antibody, as a treatment for EoE has highlighted how crucial these mechanisms are for disease persistence [4]. However, the oesophageal epithelium is also central to EoE pathophysiology both through primary mechanisms and the action of immunological mediators that disrupt homeostatic and barrier functions [5, 6, 7]. Basal cell hyperplasia, impaired epithelial cell differentiation, loss of barrier integrity, and secretion of alarmins (e.g., TSLP, IL‐33) are all features of the inflamed EoE epithelium [8, 9]. These findings emphasise the importance of the oesophageal epithelium as a key tissue that is integral to the aetiology of EoE.

In recent years, researchers have established a better understanding of the immune landscape in EoE. These studies led to the discovery of interferon (IFN) responsive gene signatures in paediatric and adult patients. Specifically, IFNα and IFNγ‐responsive gene pathways have been shown to be upregulated in the context of EoE [10]. The relevance of this observation was supported by the identification of IFNγ‐producing immune cells in the circulation and oesophageal mucosa of EoE patients [3, 11, 12]. As IFNs are pleiotropic cytokines with a multitude of pro‐inflammatory and regulatory immune functions, these findings open avenues of clinical investigation to further develop efficient and complete healthcare strategies to treat EoE patients.

Given the known roles of IFNγ in the regulation of major histocompatibility complex (MHC) II [13, 14], we hypothesised that IFNγ, derived from T cells or other sources, may regulate MHCII expression on oesophageal epithelial cells (EECs). Indeed, one study described a human oesophageal epithelium cell line that expressed human leukocyte antigen (HLA)‐DR after treatment with IFNγ [12]. Epithelial cell‐specific MHCII expression has also been described in diverse tissues including the small intestine [15, 16], lung [17, 18], and lymphatic endothelial cells [19]. Though it is highly context‐dependent, there appears to be a consensus that epithelial cell MHCII is poised to tightly regulate local T cells and prevent overamplified immune responses at sites of inflammation [20, 21]. These findings highlight the importance of epithelial MHCII in the regulation of immune responses. However, the contribution of EEC‐MHCII to oesophageal immune responses and inflammatory disease processes is currently unknown.

For this study, we sought to determine the dynamic regulation and function of MHCII in the context of EoE. To do so, we tailored a scientific approach using samples from both human patients and a previously established, clinically relevant mouse model of acute, food antigen‐driven EoE‐like inflammation [9]. Using these tools, we have found a novel role for IFNγ in regulating EEC‐MHCII expression and EoE‐like inflammation.

## 2 Methods

### 2.1 Single Cell RNA Sequencing of Human Oesophageal Epithelium

Single‐cell RNA‐sequencing (scRNA‐seq) data from Morgan et al. [22], and Rochman et al. [23], was obtained from GSE175930 and GSE201153, using all 2 control, 11 active EoE, and 7 remission EoE samples from these datasets. The raw count matrix with barcode and feature information for each sample was loaded and transformed to a Seurat v4.2.0 object. We accounted for dataset‐specific differences using different parameters for the QC and normalisation for each dataset. We set the gene count detection threshold value based on the raw reads and number of genes detected in each sample. For the data from Rochman et al., we selected cells in which the total UMI was between 700 and 30,000 and the number of identified genes was between 500 and 6000. For the data in Morgan et al., the total UMI was between 800 and 30,000, and the number of identified genes was between 500 and 8000. Cells with less than 200 detected genes and genes expressed in 3 or fewer cells were excluded from the analysis. In addition, cells with a high number of transcripts consisting of mitochondrial genes were excluded. The DecontX [24] package was used to computationally estimate and remove RNA contamination in individual cells. Finally, the DoubletFinder [25] package was used to predict and remove doublets from the dataset.

Seurat integration workflow was used to integrate the top 3000 variable genes across cells from different samples. After integration, epithelial cells were extracted based on a set of epithelial marker genes (*KRT14*, *COL17A1*, *KRT5*, *SOX2*, *KRT13*). Principle component analysis and UMAP dimensionality reduction procedures were then performed on the epithelial cells. Clustering was performed with a Shared Nearest Neighbour graph by first determining the 20 nearest neighbours for each cell. MHCII gene expression was compared across three categorical conditions: control, active EoE, and remission EoE. Pseudobulk RNA‐seq analysis was performed for differentially expressed gene (DEG) analysis on active EoE versus control, and remission EoE versus control. Specifically, raw gene expression counts were aggregated based on different cell types within each sample. Re‐organised gene expression counts were processed using DESeq2, including batch correction and normalisation. DEGs were determined as log_2_(fold change) > 1.

### 2.2 Mice

All experiments were performed in 8 to 12‐week‐old female mice, unless otherwise stated in the figure legends, and mice were bred in the Children's Hospital of Philadelphia (CHOP) specific pathogen‐free animal facility. C57BL/6J (strain #000664), B6.129S2‐*H2* ^*dlAb1*‐*Ea*^/J (*Mhcii*−/−, strain #003584), B6.129S7‐Ifngr1tm1Agt/J (*Ifngr1*−/−, strain #003288), and B6.129X1‐*H2*‐*Ab1* ^*b*‐*tm1Koni*^/J (*I*‐*Ab* ^*fl*/fl^, strain #013181) mice were purchased from Jackson Laboratories. *CIITA*‐*pIV*−/− K14 [26, 27] mice were bred on a C57BL/6J background and provided by Dr. Laurence C. Eisenlohr. ED‐L2 Cre transgenic mice were bred on a C57BL/6J background and provided by Dr. Jonathan Katz [28]. The Institutional Animal Care and Use Committee of the CHOP Research Institute (Protocol 19‐001324) approved all animal experiments.

### 2.3 Acute Model of Food Antigen‐Dependent Eosinophilic Esophagitis

#### 2.3.1 Sensitization Phase

Control and EoE mice were briefly anaesthetised with isoflurane and treated topically on both pinnae with 2 μM Calcipotriol/MC903 (Tocris Bioscience, 2700, diluted in 100% ethanol) daily for 10–12 days. Additionally, EoE mice were topically treated daily with 100 μg of ovalbumin protein (Sigma‐Aldrich, A5503) dissolved in PBS, while control mice were treated with PBS alone. Mice were rested on days 13 and 14.

#### 2.3.2 Challenge Phase

On days 15 and 17, mice were intragastrically gavaged with 50 mg of ovalbumin dissolved in DI H_2_O. Concurrently, mice had ad libitum access to water with 1.5 g/L of ovalbumin. On day 18, mice were euthanized using CO_2_, and euthanasia was confirmed using cervical dislocation.

### 2.4 Mouse Oesophagus Harvest and Processing for Single Cell Suspensions

Esophagi were harvested and placed in ice‐cold FACS buffer (PBS buffer with 5% FBS, 1× Penicillin/Streptomycin, 0.5 mM EDTA). For enzymatic digestion, the esophagi were minced on Petri dishes using razor blades. The minced tissue was then added to 5 mL of serum‐free RPMI with 1 mg/mL Collagenase/Dispase (Roche) and incubated at 37°C in a rotating VWR hybridization oven for 30 min. After incubation, the media with digested oesophageal tissue was passed through a 70‐μm filter into a 50‐mL conical tube. Any remaining tissue was mashed over the filter using the bottom end of a syringe, and the filter was subsequently washed with 10 mL of FACS buffer for a final volume of 15 mL. Tubes containing samples were kept on ice and subsequently centrifuged at 800× *g* at 4°C. After centrifugation, supernatants were discarded and pelleted cells were resuspended in 1 mL of FACS buffer in preparation for cell counting. A fraction of cells was diluted 1:10 in Trypan Blue and counted using a TC20 Automated Cell Counter 2.058 (Bio‐Rad).

### 2.5 Flow Cytometry

Oesophageal single cell suspensions were stained with Live Dead Blue (Thermo Scientific, L34962) diluted 1:1000 in PBS for 15 min at 4°C in the dark. Next, cells were washed with FACS buffer and centrifuged at 800× *g* at 4°C for 5 min, followed by resuspension and incubation in purified rat anti‐mouse CD16/CD32 Fc Block (BD Bioscience, 553142) diluted 1:200 in FACS for 15 min. Afterward, cells were washed with FACS buffer and incubated for 30 min at 4°C in the dark with surface marker specific fluorochrome‐conjugated antibodies (1:500 dilution in FACS buffer). Cells were then washed with FACS, centrifuged at 800× *g* at 4°C for 5 min, and resuspended in FACS buffer for flow cytometric analyses. All flow cytometry data were generated using either a LSR Fortessa with FACS Diva software (BD) or a Cytek Aurora with SpectroFlo 3 software. Key experimental findings were consistent between cytometers. All cytometers were maintained by the CHOP Flow Cytometry Core. Downstream data analyses were conducted using FlowJo software (v10.10, FlowJo LLC).

Gating strategies for the identification of cells of interest are outlined in Figure S2. To determine where gates should be placed for analysis, fluorescence minus one (FMO) controls were used for each fluorochrome. An inclusive cells gate was drawn based on forward and side scatter. Dead cells were excluded by drawing a Live Dead Blue negative gate. Eosinophils were identified as CD45^+^ (BD, BUV395, Clone 30F11), CD11b^+^ (Biolegend, BV785, Clone M1/70), CD11c^−^ (BD, APC‐Cy7, Clone HL3), Siglec‐F^+^ (BD, BV650, Clone E50‐2440), SSC^high^. Oesophageal epithelial cells were identified as CD45^−^, EpCAM^+^ (Thermo Fisher Scientific, PE, Clone G8.8). Additional cell types examined included dendritic cells (CD45^+^, CD11c^+^) and B cells (CD45^+^, CD19^+^). Additional markers of interest included MHCII (BD or Biolegend, BV711 or APC, respectively, Clone M5/114.15.2) and the co‐stimulatory molecules CD80 (Biolegend, BV711, Clone 16‐10A1) and CD86 (Biolegend, BV510, Clone GL‐1).

### 2.6 Human Oesophageal Epithelial Cell Organoid Culture, Cytokine Treatment, IHC and RNA‐Seq

Telomerase‐immortalised normal human oesophageal keratinocyte cell line, EPC2‐hTERT [29], was grown in standard low‐calcium keratinocyte serum‐free media (KSFM) containing 0.09 mM Ca^2+^ (Gibco), 1 ng/mL recombinant epidermal growth factor (rEGF), 50 μg/mL bovine pituitary extract (BPE) and 100 U/mL Penicillin–Streptomycin (Thermo Fisher Scientific) in a 37°C incubator with 5% CO_2_. High calcium KSFM (1.8 mM Ca^2+^) media was used to induce epithelial cell maturation. EPC2‐hTERT oesophageal organoids were prepared as previously described [30, 31]. Briefly, 2 × 10^3^ EPC2‐hTERT cells were mixed in 50 μL of Matrigel (Corning) and seeded into individual wells of 24‐well plates. Once the Matrigel solidified, 500 μL of high Ca^2+^ KSFM were added to each well. The 3D organoids were grown for 11 days at 37°C in 5% CO_2_. On days 7 and 9, the organoids were treated with 5 ng/mL of IFNγ (Sigma‐Aldrich). On day 11, organoids were isolated from the Matrigel matrix using vigorous pipetting and vortexing in DPBS (Gibco).

For immunohistochemistry (IHC), organoids were isolated and then fixed in 4% paraformaldehyde overnight. Next, they were embedded in a mixture of 2% agarose and 2.5% gelatin for paraffin embedding. The organoid sections were processed for histologic analyses by the Penn Vet Comparative Pathology Core and stained with anti‐human HLA‐DR for IHC.

For RNA sequencing, organoids were isolated, washed, and lysed in TRI Reagent. RNA was extracted using RNeasy Mini Kit (Qiagen) according to the manufacturer's protocol. RNA libraries were prepared using NEBNext Ultra II RNA Library Prep Kit for Illumina. Paired‐end sequencing was performed on Illumina HiSeq 2500 and generated data was deposited in NCBI GEO [32] under accession number GSE234424. The quality of the sequencing data was assessed with FastQC 0.14.1 [33] and MultiQC 1.10 [34] Trimmomatic 0.39 [35] was used to trim low‐quality reads and remove adapters, followed by pseudo‐alignment and quantification against GRCh38.p13 using kallisto 0.46.0 [36]. The reads were converted to counts per million (CPM), and genes with low abundance were excluded following log_2_ normalisation. Weighted trimmed mean of M‐values (TMM) normalisation was then implemented using edgeR 3.38.4 [37]. To visualise the expression patterns of antigen processing and presentation of gene signatures in unstimulated and IFNγ‐stimulated organoids, a heatmap was generated using the R package, gplots 3.1.3. The genes were extracted from the ‘hsa04612 KEGG pathway’ from the following URL: https://www.gsea‐msigdb.org/gsea/msigdb/cards/REACTOME_MHC_CLASS_II_ANTIGEN_PRESENTATION. Further, differential gene expression was analysed using limma 3.52.4 and a pathway visual depicting log_2_FoldChange in IFNγ‐stimulated compared to unstimulated organoids for KEGG PATHWAY (https://www.genome.jp/pathway/hsa04612) was created using the R package pathview 1.40.0.

### 2.7 In Vivo IFNγ Treatment and Blockade

For IFNγ gain‐of‐function experiments, mice were anaesthetised with isoflurane and treated with 1 × 10^5^ U of recombinant carrier‐free IFNγ (Biolegend, 575308) or sterile PBS intravenously via retroorbital injection daily for 2 days. The mice were euthanized on day 3, and their esophagi were harvested for flow cytometric analyses. To test how IFNγ influences our food antigen‐dependent EoE model, mice were treated with 1 × 10^5^ U of recombinant carrier‐free IFNγ on days 16 and 17 of the model, as previously described [17]. Mice were euthanized on day 18, and their esophagi were harvested for flow cytometric analyses.

For blockade of IFNγ during the EoE protocol, EoE mice were injected intraperitoneally on days 14 and 16 with 100 μg of αIFNγ blocking antibody (Bio X Cell, BE0055) or IgG1 α‐horseradish peroxidase (Bio X Cell, BP0088) as an isotype control.

### 2.8 Primary Human Oesophageal Epithelial Cell Culture

Biopsies from control and EoE subjects were collected into keratinocyte serum‐free media (KSFM; Thermo Fisher Scientific, USA) on ice. The biopsies were enzymatically digested via incubation with 10 U/mL dispase for 10 min at room temperature (RT). Following digestion, the biopsies were washed with phosphate‐buffered saline (PBS) and further treated with 0.05% trypsin at 37°C in a thermomixer at 800 RPM for 10 min.

To neutralise the trypsin activity, a trypsin inhibitor (Sigma‐Aldrich) was added to the dissociated tissues. The cell suspension was then filtered through a 70‐μm cell strainer, followed by centrifugation at 400× *g* for 5 min. The resulting cell pellet was resuspended in KSFM supplemented with 0.09 mM Ca^2+^, 1 ng/mL recombinant human epidermal growth factor, 50 μg/mL bovine pituitary extract (BPE), and 100 U/mL penicillin and streptomycin. Cells were cultured at 37°C in a humidified 5% CO₂ incubator until they reached 70%–80% confluency. Once the desired confluency was achieved, the cells were seeded into 24‐well plates and used for DQ‐OVA assays (methods shown below).

### 2.9 DQ‐OVA Incubation

The DQ‐OVA assay was performed as previously described [17]. For mouse samples, primary mouse oesophageal single cell suspensions were plated on a round bottom 96‐well plate with R10 media (RPMI +10% FBS +1% Penicillin/Streptomycin +2 mM L‐glutamine +1 mM HEPES +55 μM 2‐mercaptoethanol) and either 0 or 10 μg of DQ‐OVA (Thermo Fisher, D12053) at 4°C or 37°C for 2 h. After incubation, cells were washed with PBS and stained for flow cytometric analysis.

For the DQ‐OVA assay in primary human oesophageal epithelium, cells from a non‐EoE Control or an EoE patient were seeded in 24‐well plates as described above and incubated with 0 or 10 μg of DQ‐OVA (Thermo Fisher, D12053) at 4°C or 37°C for 2 h. After incubation, cells were washed with PBS and stained for flow cytometric analysis.

### 2.10 Histology

Histology was performed by the Comparative Pathology Core at the University of Pennsylvania School of Veterinary Medicine. For mouse esophagi, tissues were stored in scintillation vials that contained 5 mL of 4% paraformaldehyde until paraffin embedding, sectioning, and haematoxylin and eosin staining. Microscopic images were obtained using a Zeiss Widefield Microscope in the Cell and Developmental Biology Core laboratory at the University of Pennsylvania. Images were analysed using Fiji ImageJ.

### 2.11 Statistical Analyses

All statistical analyses were performed using GraphPad Prism 10 software. The statistical tests used for individual experiments are listed in their respective figure legends. For all experiments, the normality of data distribution was tested using the following built‐in tests: Anderson–Darling, D'Agostino–Pearson omnibus, Shapiro–Wilk and Kolmorogov–Smirnov. If the data failed at least one of these normality tests, non‐parametric statistical tests were performed. Datasets with two groups were analysed using an unpaired Student's *t*‐test (if data were normally distributed) or a Mann–Whitney test (if data were not normally distributed). Datasets with more than 2 groups and only 1 contributing independent variable were analysed using ordinary one‐way ANOVA with an uncorrected Fisher's LSD Test (normally distributed) or Kruskal–Wallis with an uncorrected Dunn's test (not normally distributed). One exception was the ANOVA with a corrected Tukey's multiple comparisons test used for the analysis of oesophageal biopsies in Figure 2 to account for the variability between patient samples. For the DQ‐OVA assay, since there are more than 2 experimental groups and 2 contributing independent variables, we performed a two‐way ANOVA with a Sidak's multiple comparisons test. Statistically significant differences are any comparisons that have a *p*‐value of 0.05 or less and are notated with asterisks (*) in figures and legends. Non‐statistically significant comparisons are notated with either the *p* value or ‘ns’ (not significant).

## 3 Results

### 3.1 Human Oesophageal Epithelial Cells Express HLA‐Class II‐Related Transcripts

**FIGURE 1:**
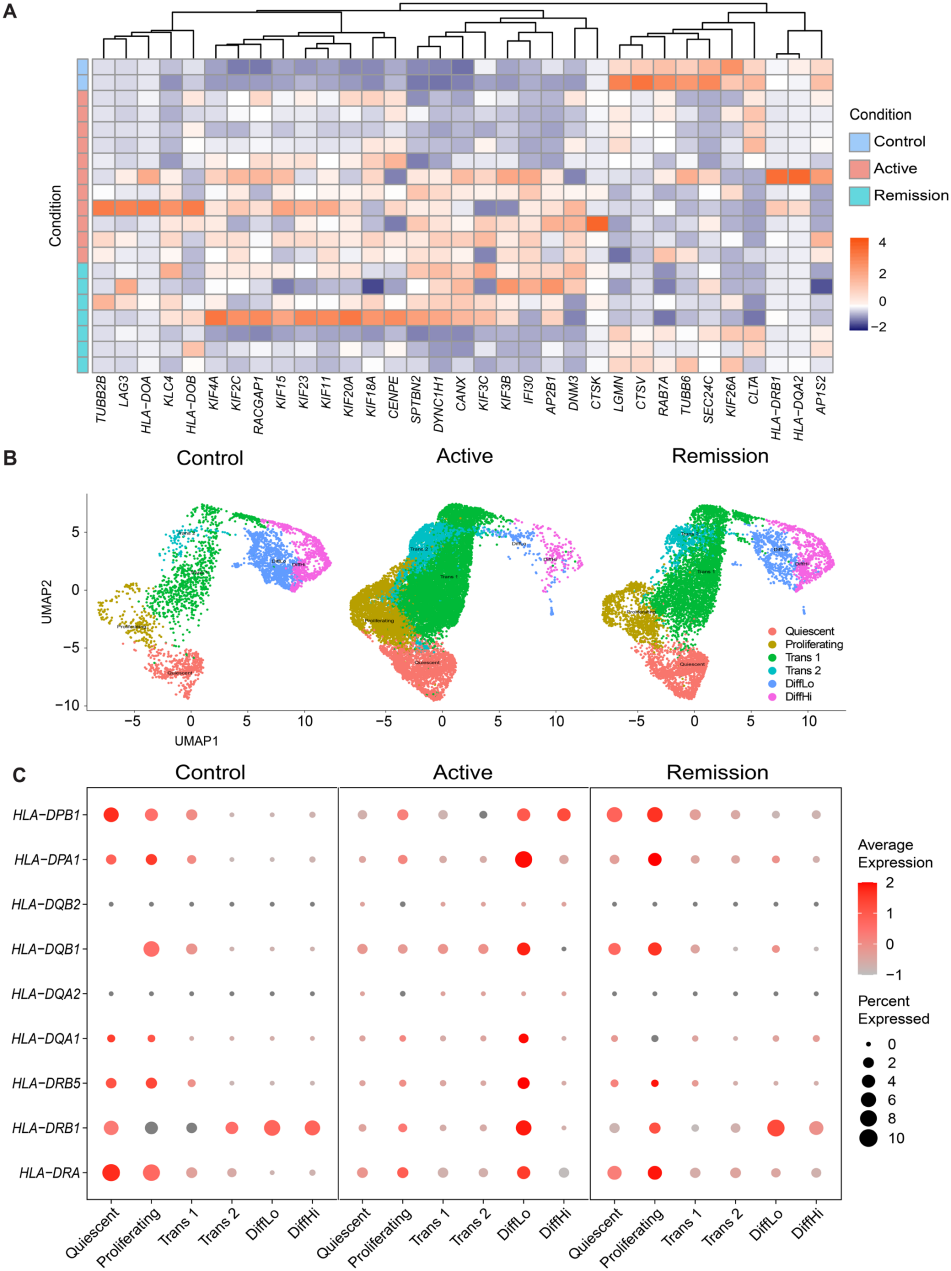
Human oesophageal epithelial cells express HLA‐Class II during eosinophilic esophagitis. (A) Heat map of HLA‐Class II pathway related genes from Control (*n* = 2), Active EoE (*n* = 11), and Remission EoE (*n* = 7) subjects. (B) Clusters of oesophageal epithelial cell subsets from Rochman et al. and Morgan et al. isolated from Control, Active EoE, and Remission EoE subjects. (C) Gene expression dot plot of HLA‐Class II alleles on oesophageal epithelial cell subsets as described in Rochman, et al. in Control, Active EoE, and Remission subjects.

To determine HLA‐Class II expression by EECs during EoE, we integrated and analysed publicly available scRNA‐seq datasets of epithelial cells isolated from oesophageal biopsies of control (*n* = 2), active EoE (*n* = 11), or remission EoE (*n* = 7) subjects [22, 23]. EECs from subjects with active or remission EoE displayed higher expression of gene transcripts related to HLA‐Class II antigen processing and presentation when compared with EECs from control subjects (Figure 1A). To better resolve gene expression among EEC subsets, we partitioned the epithelium into subsets as described in Rochman et al. (Figure 1B) [23], based on expression of population‐defining markers (Figure S1). We noted that the expression of HLA‐Class II transcripts resided predominantly in basal epithelial cell subsets (from Quiescent to Trans 1) in control subjects, shifted to differentiated subsets during active disease (DiffLo & DiffHi), and then reverted to the baseline expression pattern during disease remission (Figure 1C).

### 3.2 Mouse Food Antigen‐Dependent EoE Is Associated With Upregulated Epithelial Cell‐MHCII Expression

**FIGURE 2:**
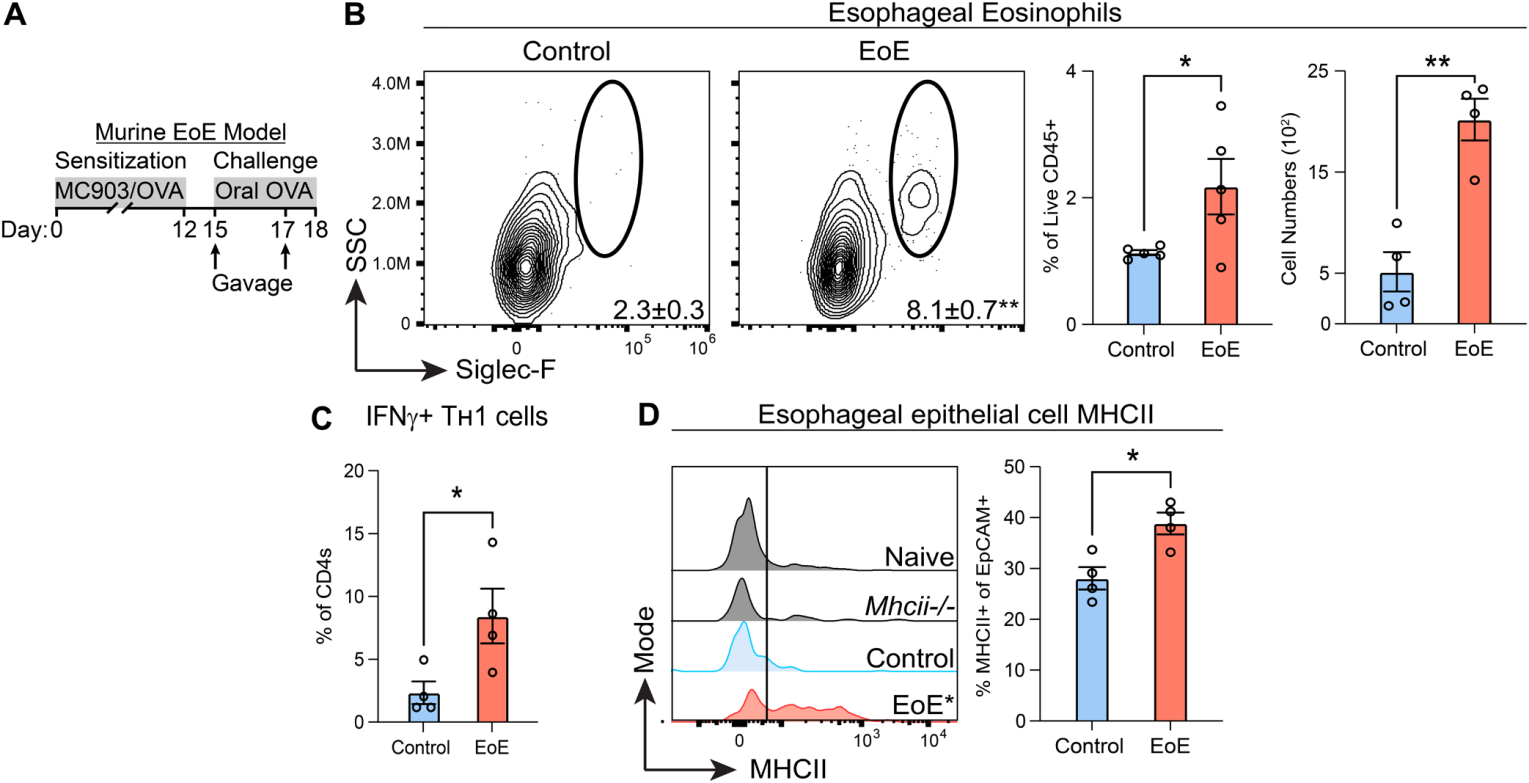
Food antigen‐dependent EoE is associated with increased FoxP3+ regulatory T cells and upregulated epithelial cell MHCII expression. (A) Schematic of murine model of EoE‐like inflammation. Control mice were treated with MC903 + PBS and experimental mice were treated with MC903 + OVA epicutaneously. (B) Representative flow plots depicting CD11b+CD11c− oesophageal eosinophilia by FACS in Control versus EoE mice, and quantification of oesophageal eosinophils as a percentage of Live CD45+ cells and cell numbers. Numbers in plots represent eosinophils as a percentage of CD11b+CD11c− (parent) ± SEM. *n* = 4–5/arm. Student's *t*‐test was used for statistical analysis of Control versus EoE. **p < 0.01. Data are presented as the mean ± SEM. *n* = 4–5/arm. Student's *t*‐test was used for statistical analysis of Control versus EoE.*p < 0.05, **p < 0.01. (C) Quantification of IFNγ+ T_H_1 cells as a percentage of CD4+ T cells in Control or EoE mice. (D) Representative histograms for MHCII expression on CD45−, EpCAM+ oesophageal epithelial cells (left), and quantification of MHCII+ expression as a percentage of EpCAM±SEM (right). Student's *t*‐test was used for statistical analyses. *p < 0.05; **p < 0.01. Representative of ≥ 3 experiments.

To study the role of EEC‐MHCII in food antigen‐dependent EoE, we turned to a previously established mouse model of acute, food antigen‐driven oesophageal eosinophilia (Figure 2A) [9]. We utilised flow cytometry to evaluate immune and epithelial cells (Figure S1). This model resulted in the oesophageal infiltration of eosinophils in experimental mice (Figure 2B), which was also observed by histology (Figure S3). Like in human patients [10], mice subjected to food antigen‐dependent EoE had increases in oesophageal IFNγ+T_H_1 cells (Figure 2C). Further, EEC MHCII expression and frequencies of MHCII+ EECs were higher in EoE mice compared with controls (Figure 2D). Together, these data indicate that mouse food antigen‐dependent EoE is associated with the accumulation of IFNγ+T_H_1 cells and upregulation of epithelial cell MHCII expression.

### 3.3 IFNγ Promotes the Induction of CIITA‐pIV‐Dependent MHCII Expression by Oesophageal Epithelial Cells

**FIGURE 3:**
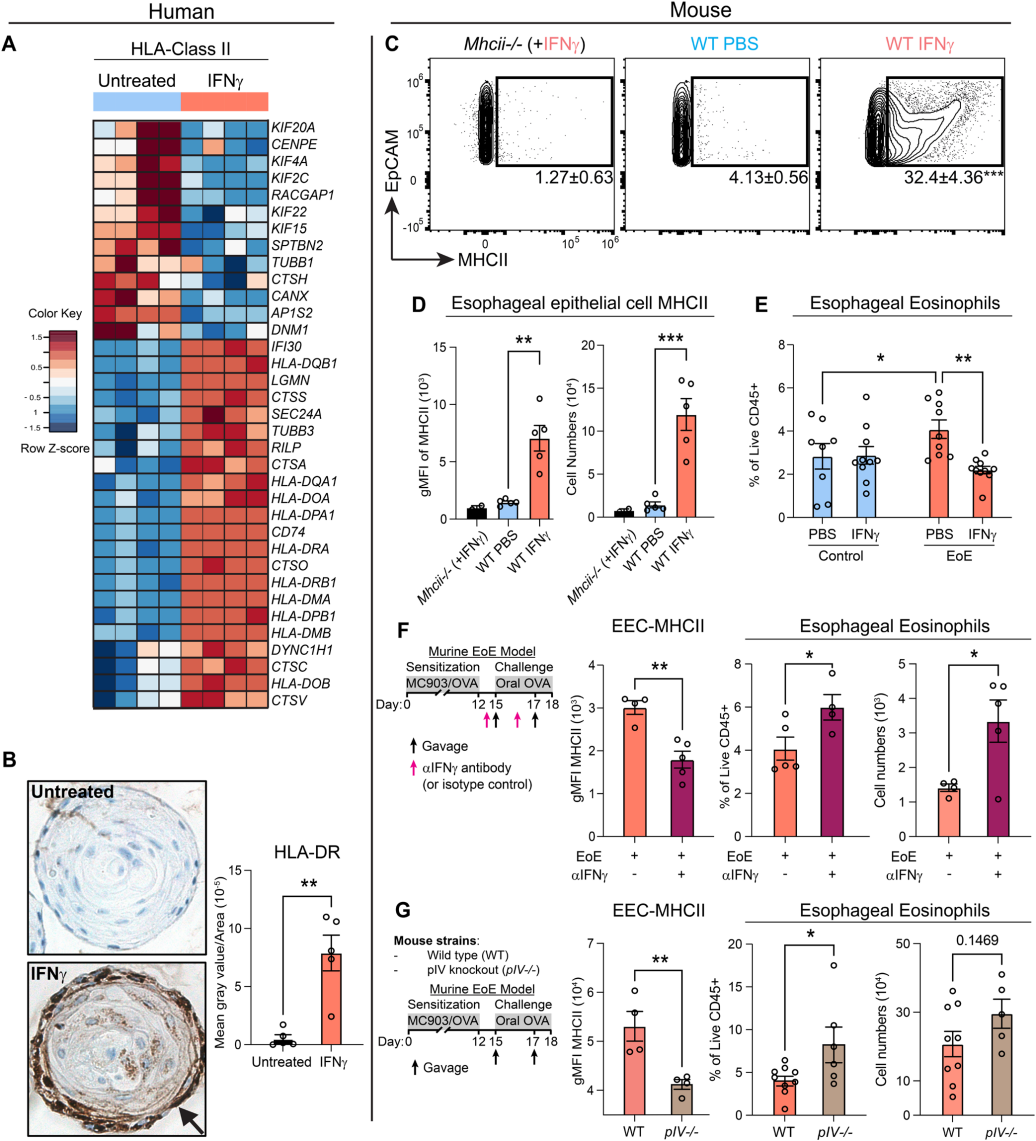
IFNγ promotes the induction of CIITA‐pIV‐dependent MHCII expression by oesophageal epithelial cells. (A) Heatmap of HLA‐Class II‐related genes in untreated or IFNγ‐treated human EPC2‐hTERT organoids. *n* = 4/arm. (B) Oesophageal organoids that are untreated or treated with recombinant IFNγ and stained for HLA‐DR. Forty times magnification. The black arrow depicts the basal cell‐like epithelial cell layer. Quantification of mean grey value/area of organoids of IHC images (right). Each circle represents one organoid (technical replicates), *n* = 5/arm. Data are presented as the mean ± SEM. Mean grey values were calculated using Fiji and normalised to area. Student's *t*‐test was used for statistical analysis. **p < 0.01. (C) Representative flow plots of CD45‐, EpCAM+ EEC‐MHCII expression after IFNγ treatment. Gated contours are MHCII+ EECs. Numbers in flow plots represent MHCII+ cells as a percentage of EpCAM+ cells (parent gate) ± SEM. (D) Quantification of MHCII as gMFI and MHCII+ cell numbers from (C). *n* = 2–5/arm. Student's *t*‐test was used for statistical analysis of PBS versus IFNγ. **p < 0.01, ***p < 0.0.001. Representative of ≥ 3 experiments. (E) Quantification of eosinophils as a percentage of Live CD45+ cells in Control or EoE mice treated with or without IFNγ during the challenge period. *n* = 8–10/arm. Two‐way ANOVA with a Fisher's LSD test was used for statistical analysis. *p < 0.05, **p < 0.01. Pooled from 2 independent experiments. (F) Schematic for murine EoE model with intraperitoneal injections of αIFNγ (or isotype control) prior to and during the challenge phase (left) and quantification of MHCII+ as gMFI and eosinophils as a percentage of Live CD45+ cells ± SEM (right). *n* = 4–5/arm. Student's t‐test was used for statistical analysis between EoE versus EoE+αIFNγ. *p < 0.05. Representative of 3 independent experiments. (G) Schematic for EoE model on WT or CIITA‐pIV knockout mice (left) and quantification of MHCII+ as gMFI and eosinophils as a percentage of Live CD45+ cells and cell numbers ± SEM (right). *n* = 4/arm. Student's *t*‐test was used for statistical analysis between WT versus *pIV*−/−. *p < 0.05, **p < 0.01. Representative of ≥ 3 experiments.

We next sought to understand the immunologic and transcriptional regulation of HLA Class II expression by human and mouse EECs. First, we treated human EPC2‐hTERT EEC organoids with IFNγ and examined HLA‐Class II‐related transcripts and protein levels. IFNγ treatment of EEC organoids resulted in an upregulation of transcripts related to HLA‐Class II antigen processing and presentation compared to untreated controls (Figure 3A), including a marked upregulation of genes in the canonical HLA‐Class II pathway (Figure S4). IFNγ treatment resulted in increased expression of HLA‐DR by EEC organoids, with the majority of HLA‐DR expression in the basal‐like EECs (outer ring) (Figure 3B).

Next, we turned to our mouse system to investigate the regulation of EEC MHCII in vivo. Treatment of wild‐type C57BL/6 mice (WT) with recombinant IFNγ resulted in a robust induction of MHCII on EECs when compared to IFNγ‐treated *Mhcii*−/− or WT PBS‐treated mice (Figure 3C,D). To test the contribution of IFNγ to food antigen‐dependent EoE, we used a combination of gain‐ and loss‐of‐function approaches. We also observed that EoE mice treated with recombinant IFNγ had significantly reduced oesophageal eosinophilia compared to PBS‐treated EoE mice (Figure 3E). Conversely, we observed that *Ifngr1*−/− mice had significantly reduced MHCII expression at baseline compared to their WT counterparts (Figure S5A). In addition, antibody‐mediated depletion of IFNγ in WT EoE mice resulted in decreased EEC MHCII expression and exacerbated oesophageal eosinophilia compared to EoE mice that received an isotype control antibody (Figure 3F). IFNγ has been shown to stimulate MHCII expression by non‐haematopoietic cells via a tightly regulated pathway dependent on the promoter IV (pIV) region upstream of the Class II transactivator (CIITA) locus [13, 38]. To test if EEC‐MHCII expression was dependent on pIV, we induced EoE in WT or *pIV*−/− mice. We observed that MHCII expression was significantly impaired on EECs (Figure 3G), but not on B cells or CD11c^+^ dendritic cells (Figure S5B), of *pIV*−/− EoE as compared with WT EoE mice. EoE *CIITA*‐*pIV*−/− mice also displayed exacerbated oesophageal eosinophilia compared with WT EoE mice (Figure 3G). Together, these data indicate that IFNγ promotes the induction of CIITA‐pIV‐dependent MHCII expression by oesophageal epithelial cells. Further, this expression correlates with reduced food antigen‐dependent eosinophilic inflammation.

### 3.4 Oesophageal Epithelial Cells Proteolytically Cleave Ovalbumin and Express Activating Co‐Stimulatory Molecules

**FIGURE 4:**
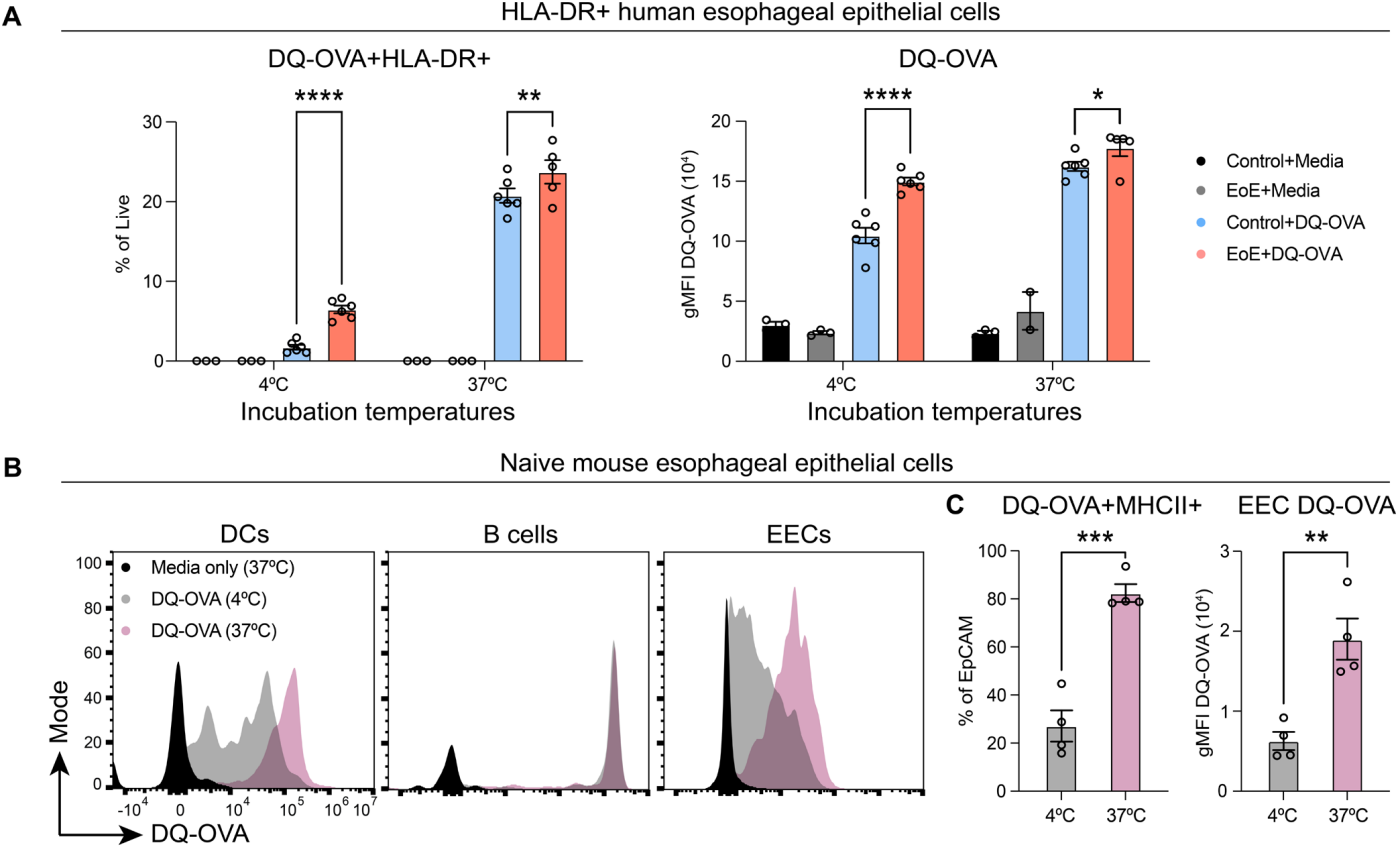
Human and mouse oesophageal epithelial cells proteolytically cleave ovalbumin. (A) Quantification of DQ‐OVA+HLA‐DR+ percentages of Live cells (left) and quantification of gMFI of DQ‐OVA (right) on primary HLA‐DR+ human EECs from either a Control or EoE patient incubated at 4°C or 37°C. Data are presented as mean ± SEM. *n* = 2–6 technical replicates/arm. Two‐way ANOVA with an uncorrected Fisher's LSD test was used for statistical analysis. *p < 0.05, **p < 0.01, ****p < 0.0001. Representative of ≥ 2 experiments. (B) Representative histograms of DQ‐OVA expression on murine DCs, B cells and EECs. (C) Quantification of DQ‐OVA+MHCII+ frequencies as a percentage of EpCAM+, and DQ‐OVA gMFI on MHCII+ murine EECs. Data are presented as mean ± SEM. *n* = 4/arm. Student's *t*‐test was used for statistical analysis. **p < 0.01, ***p < 0.001. Representative of 3 independent experiments.

Given that EECs expression of MHCII, we sought to test if they shared other functions with professional APCs. First, we tested if epithelial cells from EoE patients and non‐EoE controls could proteolyze exogenously acquired substrates by treating oesophageal epithelial cell suspensions with dye‐quenched ovalbumin (DQ‐OVA), which emits fluorescence if proteolytically cleaved. We observed temperature‐dependent increases of DQ‐OVA+ frequencies and expression in both non‐EoE control and EoE EECs (Figures 4A and S6A). Notably, we also observed that EECs from EoE patients had significantly higher DQ‐OVA+HLA‐DR+ frequencies and DQ‐OVA expression on HLA‐DR+ cells compared to non‐EoE controls (Figure 4A), while DQ‐OVA+HLA‐DR‐ EECs were reduced in frequency and displayed reduced proteolysis of OVA (Figure S6A). In addition, we tested if mouse EECs could proteolyze exogenously acquired DQ‐OVA in a manner similar to human EECs. We observed that mouse oesophageal DCs and EECs displayed temperature‐dependent increases in DQ‐OVA fluorescence (Figure 4B) that were significantly higher at 37°C (Figure S6B), while B cells displayed DQ‐OVA fluorescence irrespective of temperature. In addition, we observed significantly higher frequencies of DQ‐OVA+MHCII+ EECs, and that these MHCII+ EECs also displayed temperature‐dependent processing of DQ‐OVA+ (Figure 4C). Together, these data indicate that both human and mouse EECs display temperature‐dependent proteolytic cleavage of ovalbumin, and that human EoE EECs process the ovalbumin substrate to a higher degree than non‐EoE EECs.

### 3.5 EEC‐Intrinsic MHCII Limits Eosinophilia During Food Antigen‐Dependent EoE

**FIGURE 5:**
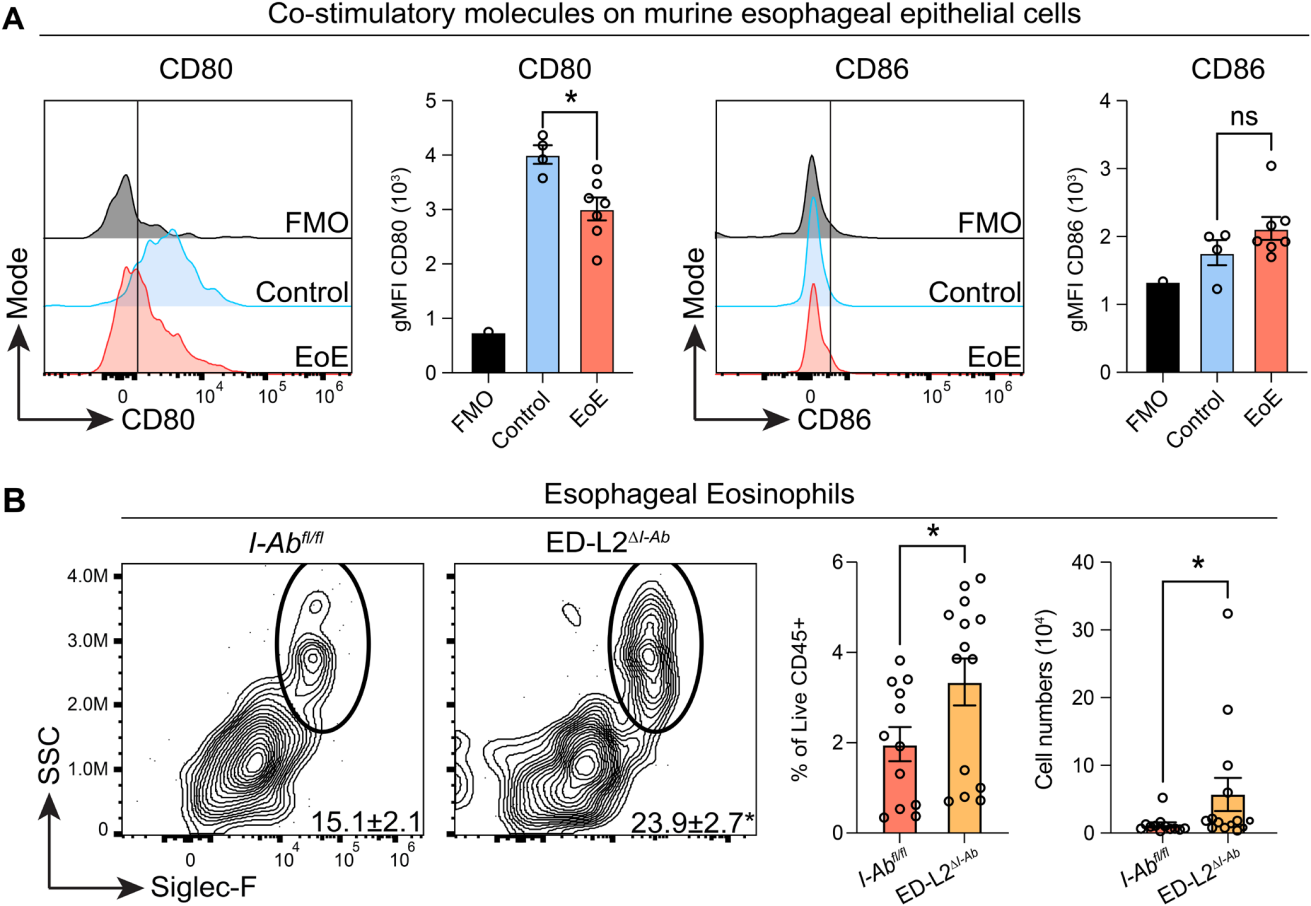
EECs express co‐stimulatory molecules, and EEC‐intrinsic MHCII limits eosinophilia during food antigen‐dependent EoE. (A) Representative histograms and graphs of gMFIs of CD80 and CD86, respectively, on CD45−, EpCAM+ EECs in Control or EoE. FMO = fluorescence minus one. Data are presented as mean ± SEM. *n* = 4–7/arm. Student's *t*‐test was used for statistical analysis. *p < 0.05, ns = not significant. Representative of 3 experiments. (B) Representative flow plots and quantification of oesophageal eosinophils in *I*‐*Ab* ^*fl*/*fl*^ and ED‐L2^*ΔI*‐*Ab*^ with EoE. Numbers in plots represent eosinophils as a percentage of CD11b+CD11c− (parent) ± SEM. *n* = 12–14/arm. Student's *t*‐test was used for statistical analysis of flow plots. Mann–Whitney test was used for statistical analysis of quantification. *p < 0.05. Representative of ≥ 3 pooled experiments.

Finally, we sought to determine the extent to which EEC MHCII modulates inflammatory features of food antigen‐dependent EoE. To do so, we first examined the expression of co‐stimulatory molecules by EECs. EECs from WT control mice expressed high levels of CD80, but not CD86 (Figure 5A). Notably, EEC CD80 expression was significantly reduced during EoE when compared to expression levels in control mice (Figure 5A). Finally, we tested the contribution of EEC‐specific MHCII to the oesophageal inflammation during EoE by crossing ED‐L2 Cre transgenic mice, which have an Epstein Barr virus lytic cycle promoter sequence with expression restricted to the oesophagus, tongue, and forestomach [28, 39], to MHCII floxed mice (*I*‐*Ab* ^*fl*/*fl*^) (EEC‐MHCII KO mice). This resulted in the reduced, but not completely ablated, expression of MHCII on oesophageal epithelial cells (Figure S7A,B). We then subjected EEC‐MHCII KO and littermate control mice to our EoE model. We observed that EoE EEC‐MHCII KO mice had significantly increased oesophageal eosinophils when compared to EoE littermate WT controls (Figure 5B). Together, these data indicate that EEC‐intrinsic MHCII limits oesophageal eosinophilia during a model of acute, food antigen‐dependent EoE.

## 4 Discussion

The contribution of T2‐inflammatory pathways to the food allergy EoE has been well described [2]. However, recent evidence has demonstrated that the EoE immunological landscape contains previously unappreciated type I and type II interferon‐regulated gene signatures [10], indicating that non‐T2 pathways are also active in this disease state. For example, a prior report documented increased IFNγ in oesophageal biopsies from EoE patients compared to biopsies from unaffected individuals or those with gastroesophageal reflux disease [12]. The authors speculated that EEC‐MHCII could be influencing EoE pathogenesis; however, the tools necessary to test this hypothesis were not available at the time. Herein, we build on these prior findings through translational studies of EoE patient tissues and advanced mouse experimental systems to dissect the mechanisms governing EEC‐MHCII expression and understand its biological significance in the context of food antigen‐dependent EoE.

Our data reveal that both human and mouse EECs express MHCII at baseline and upregulate MHCII expression during active EoE. The mRNA‐level expression of HLA alleles appears to predominate in more quiescent EECs in the steady state and during disease remission but shifts towards more differentiated EEC subsets during active disease. The spatial distribution of HLA expression in EECs is notable as it could facilitate EEC‐MHCII interaction with immune cells in the oesophageal lamina propria to promote inflammation or immune homeostasis [40]. Indeed, MHCII expression by intestinal epithelial cells (IECs) has been implicated in several immune‐mediated events including, but not limited to, responses to commensal microbes [21, 41], graft versus host disease [42], cancer [43], and maintenance of tolerance to food antigens [44]. Notable limitations of the existing human transcriptional data utilised in our analyses are that the patient cohorts were not matched between the experimental groups, and there were varying treatment regimens that were utilised to achieve clinical remission [22, 23]. Nevertheless, our new evidence that EECs express MHCII in vivo adds to the existing literature and supports the concept that the oesophagus is not just a passive structure with the sole function of transporting food to the stomach, but also a dynamic tissue with the potential to sample oesophageal luminal contents during injury or inflammation and augment immunological responses [45].

CIITA is a master controller of MHCII‐related gene expression and function [13, 46]. IFNγ is well known to promote MHCII transcription and translation in non‐haematopoietic cells by inducing Signal Transducer And Activator Of Transcription 1 (STAT1) binding to the pIV‐specific region of the CIITA locus [38, 47, 48]. This tightly controlled mechanism allows for cells of non‐immune origin to actively participate in the immune response during localised inflammation [17, 26]. Our work establishes that EECs are not an exception to this rule. In addition, our data indicate that EECs express the necessary machinery to process antigen. We also find that EECs express the co‐stimulatory molecule CD80 at baseline and that this expression is decreased in EoE. MHCII signalling in the absence of co‐stimulatory molecules has been associated with the induction of T cell anergy [49], which is a mechanism by which the epithelium may prevent overamplification of immune responses that would otherwise be detrimental. The sum of these data suggests that EECs are well poised to modulate adaptive immune responses in the oesophageal mucosa.

Consistent with EEC‐MHCII being a critical regulator of the mucosal immune response, we found that impairment of upstream mediators that promote EEC‐MHCII expression (IFNγ, CIITA‐pIV) led to exacerbated food antigen‐driven oesophageal eosinophilia. Conversely, recombinant IFNγ treatment led to reduced eosinophilia during food antigen‐dependent EoE. This evidence suggested that collectively, EEC‐MHCII may be upregulated during EoE as it performs a predominantly regulatory function, albeit an inadequate one. Indeed, our data on human EECs upregulating HLA‐DR during active EoE, and sustaining expression into disease remission, further support an immunoregulatory role for EEC‐MHCII.

To begin to test this hypothesis, we developed a novel, EEC‐specific MHCII genetic deletion system by crossing ED‐L2 Cre mice to MHCII floxed mice (*I*‐*Ab* ^*fl*/*fl*^). Disruption of EEC‐MHCII resulted in exacerbated eosinophilic inflammation, confirming that EEC‐MHCII exerts a predominantly regulatory role in this allergic disease. To our knowledge, this is the first direct examination of the role of EEC‐MHCII in oesophageal inflammation. Though we extensively studied the regulation and function of EEC‐MHCII in our food antigen‐dependent EoE systems, how it performs its regulatory role is a subject that requires further investigation. However, MHCII could directly influence the development of tolerance to food antigens by modulating T_H_2 cell responses or promoting the expansion and the anti‐inflammatory functions of regulatory T cells, as previously described for IECs [15, 44, 50].

A complementary, and not necessarily mutually exclusive, potential function for EEC‐MHCII could be the control of EEC differentiation and turnover [41, 51]. Given that one of the histological characteristics of EoE is basal cell hyperplasia [8, 52, 53], EEC MHCII governing the maturation of the oesophageal epithelium is a reasonable hypothesis. For example, cytokines derived from T helper cells regulate intestinal stem cell differentiation into mature intestinal epithelial cell subsets [51]. MHCII expression by these stem cells was proposed to be involved in this mechanism, as ablation of stem cell‐specific MHCII halted the differentiation process. Another recent report showed that IEC‐MHCII induced by segmented filamentous bacteria promoted the conversion of intestinal T helper cells into more of a ‘cytotoxic’ intraepithelial lymphocyte and showed that this event was important for epithelial cell turnover [41]. Interestingly, the DiffLo and DiffHi populations of EECs, which express high levels of HLA transcripts during active disease, are involved in the biological processes of epithelial cell differentiation and keratinization [23], supporting the hypothesis of MHCII being important for the maintenance of the oesophageal epithelial barrier. Thus, further studies of the role of EEC‐MHCII in EEC development and differentiation are warranted.

In addition, a recent study uncovered that IFNγ‐treatment of human EEC organoids dysregulates the normal function and proliferation of the oesophageal epithelium in vitro [31]. Given that type 2 cytokines, such as IL‐13, also disrupt the oesophageal barrier [5], it would be of great interest to study how both IFNγ and type 2 inflammatory signalling work in conjunction during active EoE. Our study, along with the previous reports on this topic [8, 41, 51, 52, 53], may serve as the foundation for these areas of future research.

In summary, we describe a novel role for EEC‐MHCII in controlling the eosinophilic infiltration observed in our experimental systems of food antigen‐dependent EoE, highlighting its protective role against allergic inflammation. Future research efforts may consider whether EEC HLA‐Class II expression is a prognostic marker for disease severity and treatment outcomes in EoE. Further study of EEC‐MHCII and how it modulates oesophageal immune responses is also merited, as this avenue holds promise in the pursuit of more specific therapeutic strategies to boost immune regulatory responses and improve patient outcomes.

## Author Contributions

Study design: E.M.R.‐L., L.C.E., D.A.H. Contributed analytic tools and expertise: R.L.C., M.L., Y.Z., S.D.C., C.‐A.A., R.G., J.B., A.B.M., J.M.S., M.A.R., L.C.E., D.A.H. Data analysis: E.M.R.‐L., R.L.C., M.L., Y.Z., S.D.C., C.‐A.A., D.A.H. Interpretation of results: E.M.R.‐L., R.L.C., L.C.E., D.A.H. Writing of manuscript: E.M.R.‐L., D.A.H. Reviewed and approved manuscript: all authors.

## Funding

This work was directly supported by a Faculty Development Award from the American Academy of Allergy, Asthma, and Immunology (to D.A.H.), the Children's Hospital of Philadelphia Food Allergy Pilot Award (to D.A.H.), and funds from Ira and Diana Riklis (to J.M.S.). Allergy research in the Hill laboratory is also supported by the Hartwell Foundation, the Food Allergy Fund, the American Partnership for Eosinophilic Disorders, the Children's Hospital of Philadelphia Research Institute, and the National Institutes of Health (R01HL162715). E.M.R.‐L. and R.L.C. were supported by the Immune System Development and Regulation Training Grant (5‐T32‐AI055428). The contents are those of the authors and do not necessarily represent the official views nor an endorsement of the NIH or other funders. None of the funding sources had a role in the design or conduct of the study.

## Conflicts of Interest

The authors declare no conflicts of interest.

## Supporting information


**Figure S1:** cea70205‐sup‐0001‐supinfo.pdf.

## Data Availability

The data that support the findings of this study are available from the corresponding author upon reasonable request.
