# Supplementary material for: Oesophageal Epithelial Cell‐Intrinsic MHCII Regulates Food Antigen‐Dependent Eosinophilic Esophagitis in an IFNγ‐Dependent Manner
Source: Clin Exp Allergy. 2025 Dec 22;56(2):124–36. doi: 10.1111/cea.70205 (PMC12813630; doi:10.1111/cea.70205)
Supplement: Supplementary file 1 — Figure S1: cea70205‐sup‐0001‐supinfo.pdf. [file CEA-56-124-s001.pdf]

Rodríguez-López, Clement, et al. - Supplementary Figure 1

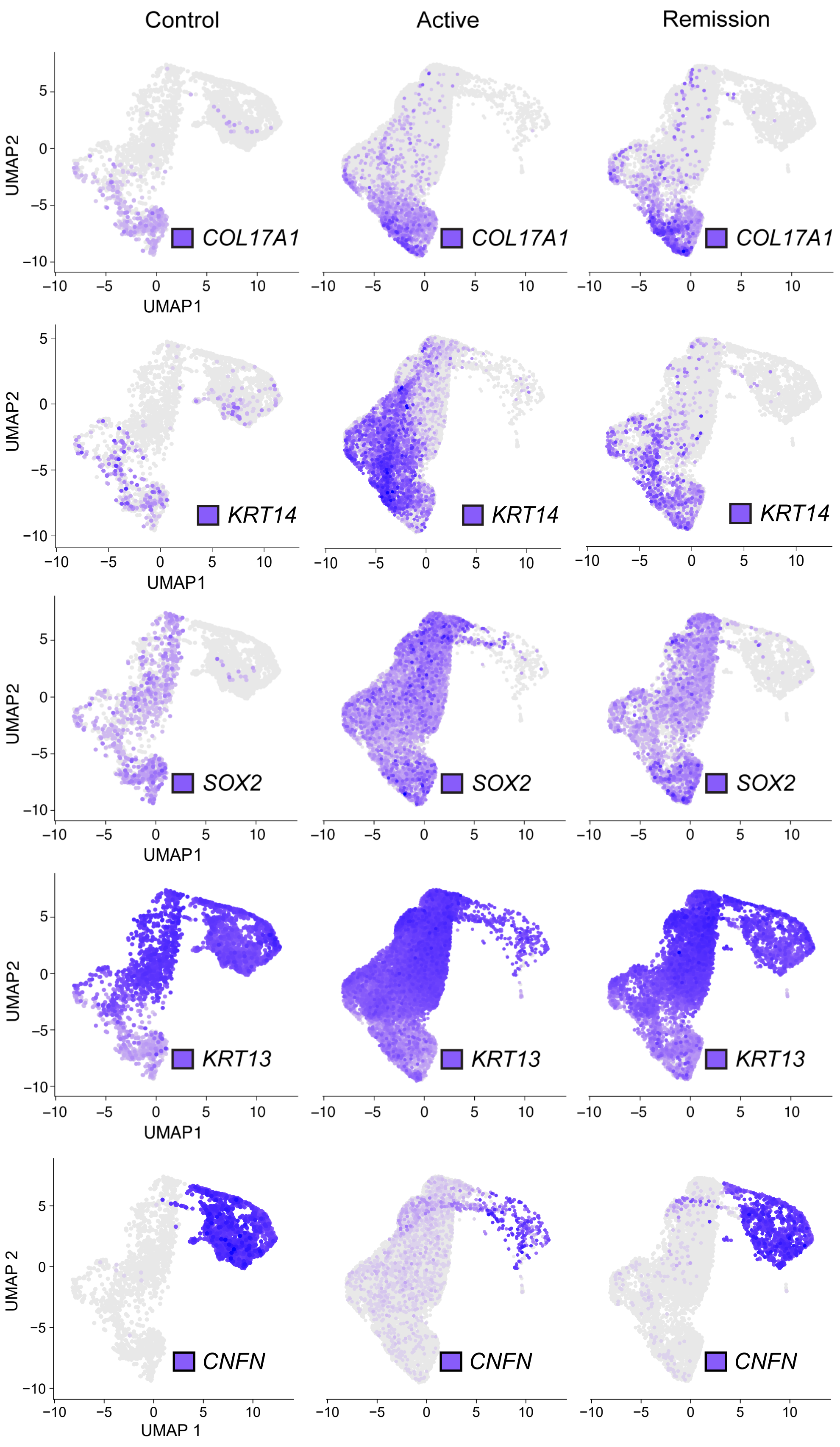

Supplementary Figure 1: Epithelial cell gene analysis of basal esophageal epithelial populations in Control, Active, and Remission EoE subjects. UMAP plots of *COL17A1*, *KRT14, SOX2*, *KRT13, and CNFN* expression in basal esophageal epithelial cells from Control, Active EoE, and Remission EoE subjects.

Rodríguez-López, Clement, et al. - Supplementary Figure 2

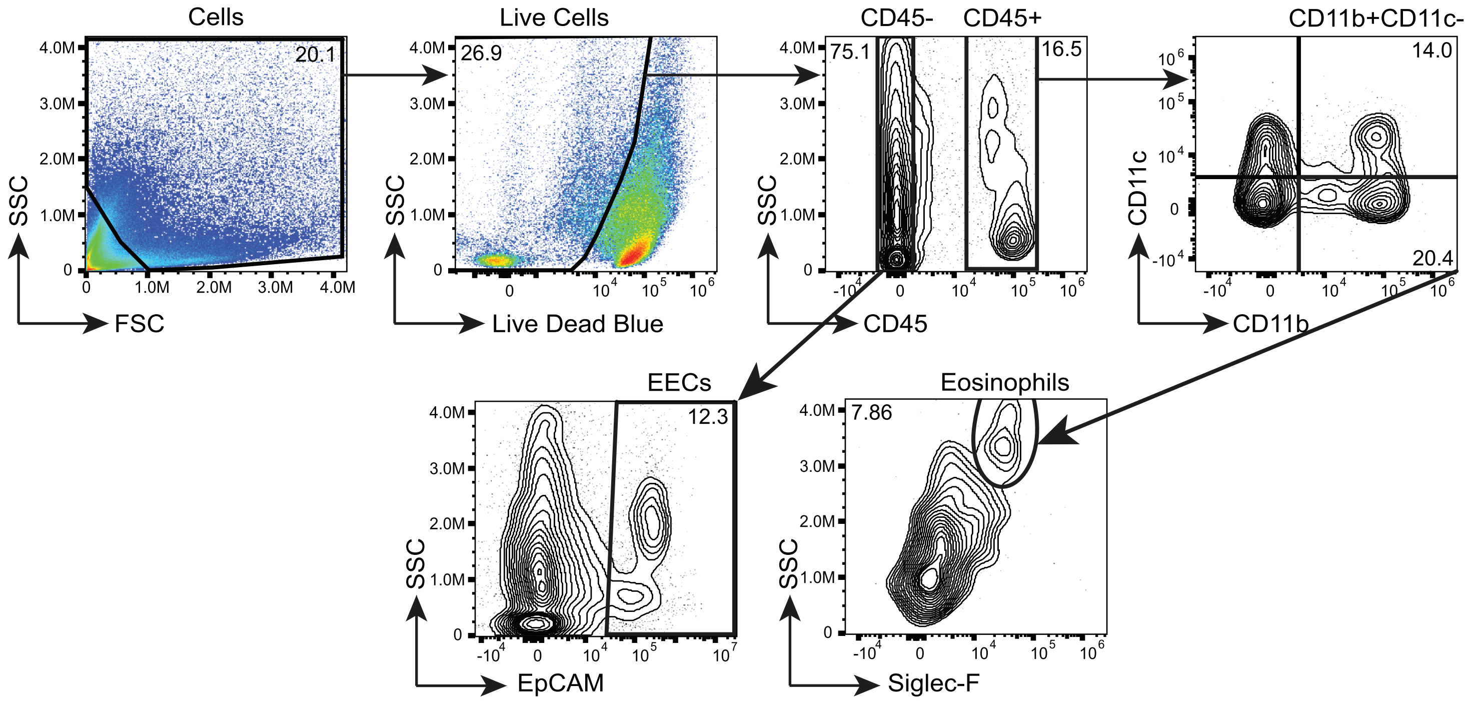

Supplementary Figure 2: Flow cytometry gating strategies for esophageal epithelial cells and eosinophils.

Rodríguez-López, Clement, et al. - Supplementary Figure 3

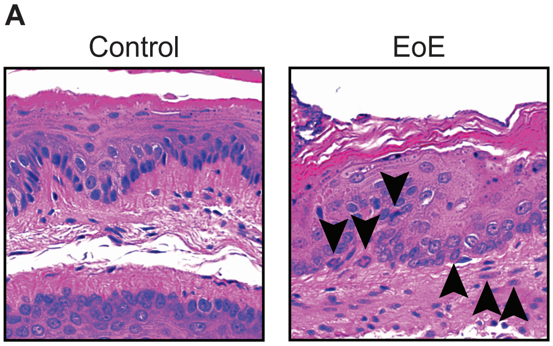

Supplementary Figure 3: Characterization of eosinophillia in a food antigen-dependent model of EoE.
(A) Representitive H&E images of mouse esophageal epithelium from Control or EoE mice. Black arrows depict eosinophils.

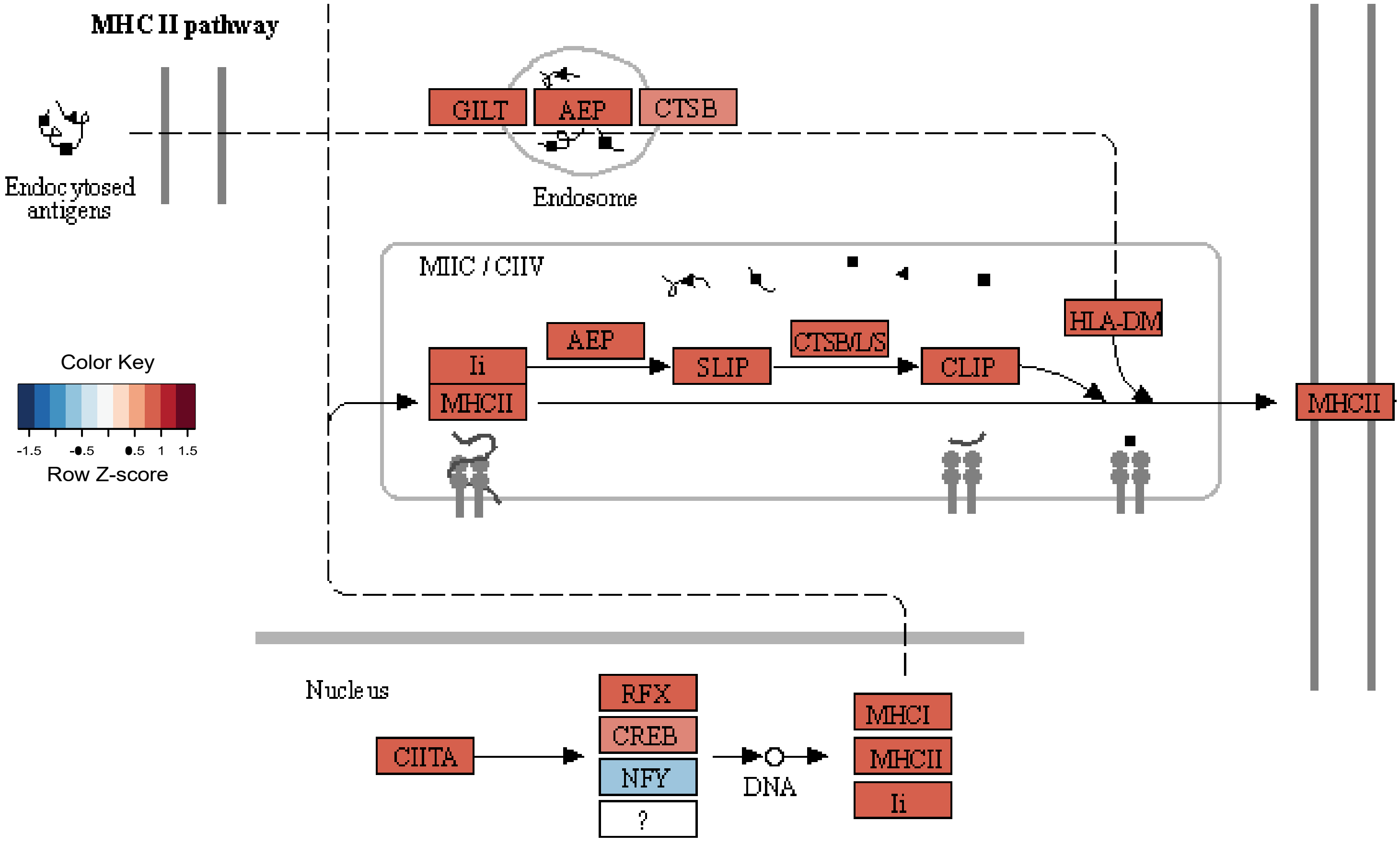

Supplementary Figure 4: Schematic of MHCII antigen presentation pathway genes upregulated by IFNγ in human esophageal epithelial cell organoids.

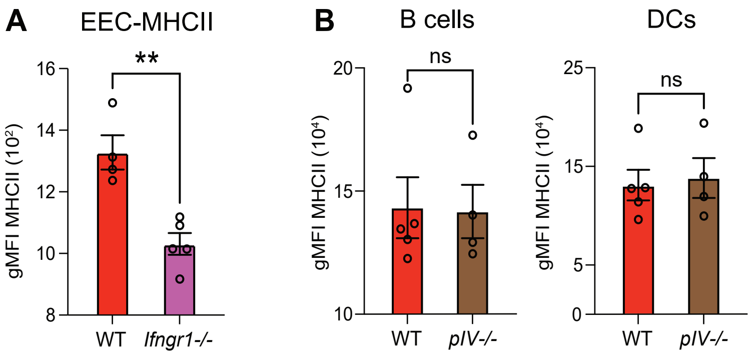

Supplementary Figure 5: EEC-MHCII expression is dependent on IFNγ signaling.
(A) Quantification of EEC-MHCII at baseline as gMFI on WT or *Ifngr1-/-* mice. Representative of 2 independent experiments. n=4-5/arm.
Student's t-test was used for statistical analysis. **p<0.01.
(B) Quantification of B cell and CD11c+ DC MHCII as gMFI on WT EoE or *pIV-/-* EoE mice. Representative of 3 independent experiments. n=4-5/arm.
Student's t-test was used for statistical analysis. ns=not significant.

Rodríguez-López, Clement, et al. - Supplementary Figure 6

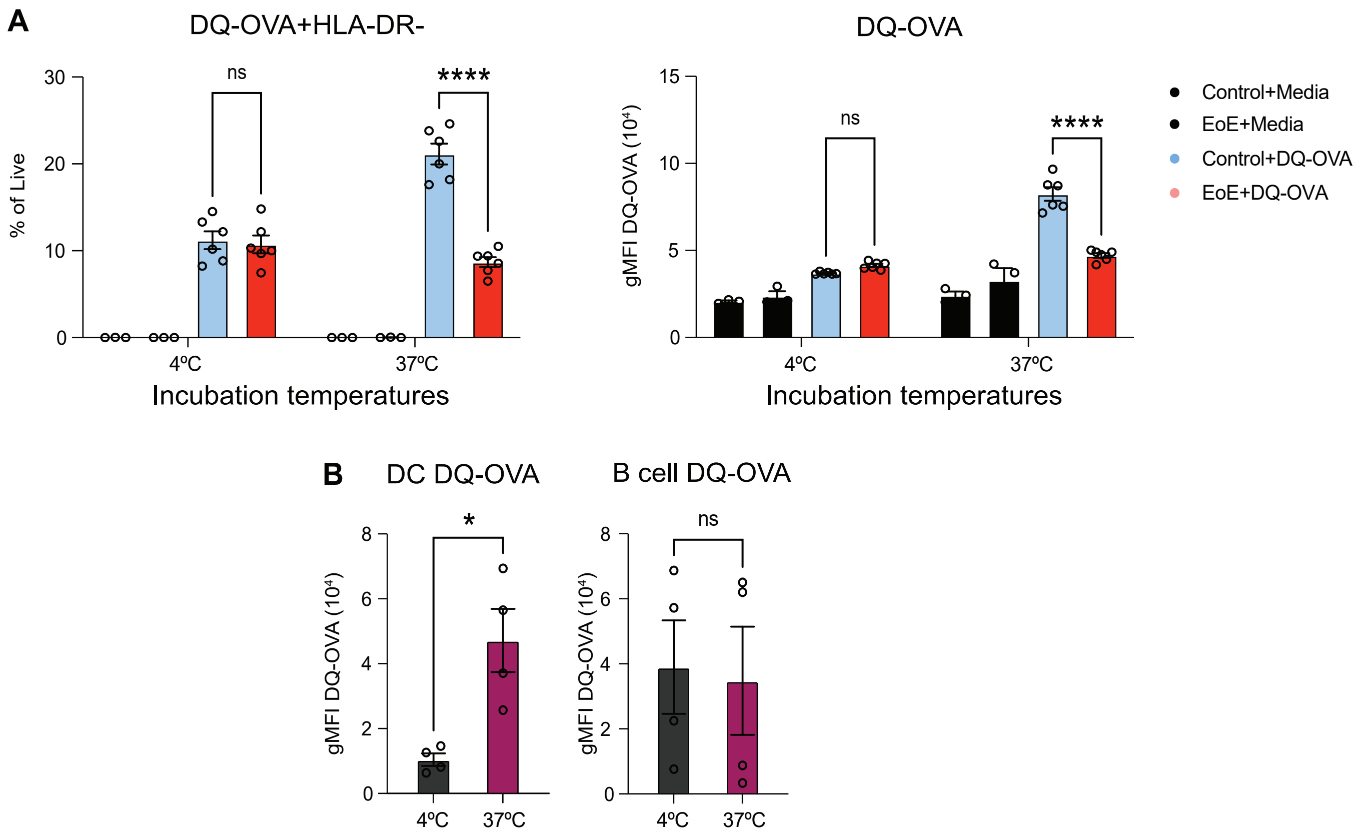

Supplementary Figure 6: DQ-OVA proteolytic processing analyses on mouse DCs and B cells, and HLA-DR- human EECs.
(A) Quantification of DQ-OVA+HLA-DR- percentages of Live cells (left) and quantification of gMFI of DQ-OVA (right) on primary HLA-DR- human EECs from either a Control or EoE patient incubated at 4°C or 37°C. Data are presented as mean±SEM. n=3-6 technical replicates/arm. Two-way ANOVA with an uncorrected Fisher's LSD test was used for statistical analysis. ****p<0.0001, ns=not significant. Representative of 2 independent experiments.
(B) Quantification of DQ-OVA gMFI on esophageal dendritic cells (DCs) and B cells. Data are presented as mean±SEM. n=4/arm.
Student's t-test was used for statistical analysis. *p<0.05, ns=not significant. Representative of 3 independent experiments.

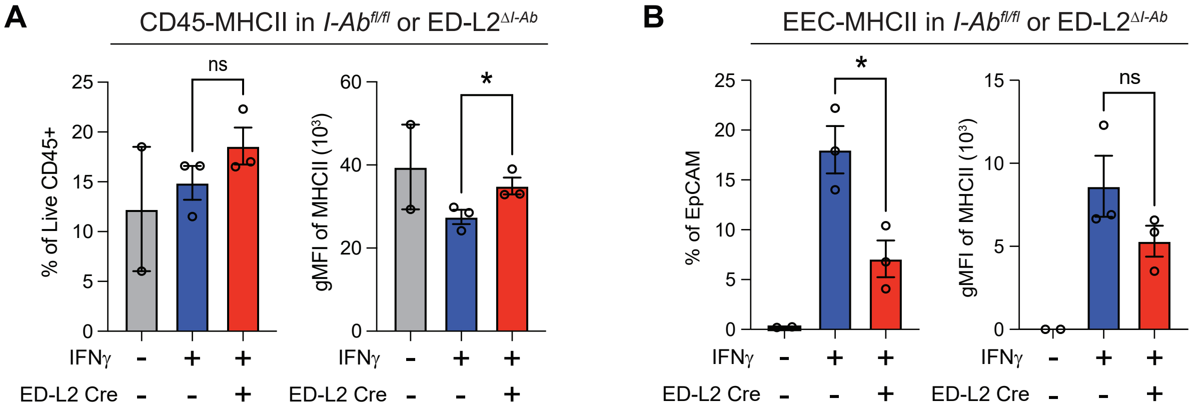

Supplementary Figure 7: ED-L2 Cre-mediated excision of the *I-Ab* allele.

(A) Quantification of CD45-MHCII as % of Live CD45 and gMFI on *I-Ab*$^{fl/fl}$ or ED-L2$^{\Delta I\text{-}Ab}$ mice treated with IFNγ. Representative of 3 experiments. n=2-3/arm Data are presented as mean±SEM. Student's t-test was used for statistical analysis. *p<0.05, ns=not significant.

(B) Quantification of EEC-MHCII as % of EpCAM and gMFI on *I-Ab*$^{fl/fl}$ or ED-L2$^{\Delta I\text{-}Ab}$ mice treated with IFNγ. Representative of 3 experiments. n=2-3/arm. Data are presented as mean±SEM. Student's t-test was used for statistical analysis. *p<0.05. ns=not significant.
